# Transesophageal Endoscopic Ultrasound Fine Needle Biopsy for the Diagnosis of Mediastinal Masses: A Retrospective Real-World Analysis

**DOI:** 10.3390/jcm11185469

**Published:** 2022-09-17

**Authors:** Daniela Assisi, Filippo Tommaso Gallina, Daniele Forcella, Riccardo Tajè, Enrico Melis, Paolo Visca, Federico Pierconti, Emanuela Venti, Francesco Facciolo

**Affiliations:** 1 Digestive Endoscopy Unit, IRCCS Regina Elena National Cancer Institute, 00144 Rome, Italy; 2 Thoracic Surgery Unit, IRCCS Regina Elena National Cancer Institute, 00144 Rome, Italy; 3 Department of Pathology, IRCCS Regina Elena National Cancer Institute, 00144 Rome, Italy; 4 Anesthesiology and Intensive Care Unit, IRCCS Regina Elena National Cancer Institute, 00144 Rome, Italy

**Keywords:** endoscopic ultrasound, fine needle aspiration biopsy, mediastinal lymph node, para-mediastinal masses

## Abstract

*Background:* Endoscopic ultrasound (EUS) plays an important role in the diagnosis and staging of thoracic disease. Our report studies the diagnostic performance and clinical impact of EUS fine needle aspiration (FNA) in a homogenous cohort of patients according to the distribution of the enlarged MLNs or pulmonary masses. *Methods:* We retrospectively reviewed the diagnostic performance of 211 EUS-FNA in 200 consecutive patients with enlarged or PET-positive MLNs and para-mediastinal masses who were referred to our oncological center between January 2019 and May 2020. *Results:* The overall sensitivity of EUS-FNA was 85% with a corresponding negative predictive value (NPV) of 56% and an accuracy of 87.5%. The sensitivity and accuracy in patients with abnormal MLNs were 81.1% and 84.4%, respectively. In those with para-mediastinal masses, sensitivity and accuracy were 96.4% and 96.8%. The accuracy for both masses and lymph nodes was 100%, and in the LAG (left adrenal gland), it was 66.6%. *Conclusions:* Our results show that, in patients with suspected mediastinal masses, EUS-FNA is an accurate technique to evaluate all reachable mediastinal nodal stations, including station 5.

## 1. Introduction

Endoscopic ultrasound (EUS) plays an important role in the diagnosis and staging of thoracic diseases. It is considered the most effective technology in visualizing and obtaining samples from mediastinal or para-mediastinal lesions [1,2,3]. Lung cancer remains the leading cause of cancer-related death worldwide, and correct disease staging, particularly in lung cancer, is essential for planning the most effective curative treatment [4,5,6,7]. Although imaging techniques such as fluorodeoxyglucose positron emission tomography (FDG-PET) with or without integrated CT scans contributes to mediastinal staging, the acceptance that PET-positive mediastinal lymph nodes are associated with malignant involvement runs the risk of excluding patients from potentially curative surgery [8,9,10]. Thus, accurate mediastinal staging requires tissue biopsies in case of either CT-enlarged or FDG-avid mediastinal lymph nodes (MLNs) [11,12,13]. Up until a decade ago, the exploration of mediastinal masses was performed using surgical approaches such as cervical mediastinoscopy, regarded for its paratracheal stations; Video-Assisted Thoracic Surgery (VATS) for the sampling of stations 5, 6, 7 and 8; and anterior left mediastinotomy for the sampling of stations 5 and 6 [14]. In the last few years, some authors have questioned the role of surgery as the standard procedure of staging MLNs [15] and have raised the advantages of preoperative staging with EUS and endobronchial ultrasound (EBUS), especially for avoiding unnecessary surgical exploration [16,17,18]. Therefore, over the last few decades, evidence-based guidelines have promoted a progressive increase in the use of these two endoscopic techniques in the diagnosis and staging of lung cancer to confirm suspected N2/N3 disease [19,20]. Non-randomized trials in selected patient populations have suggested that the sensitivities of these techniques are in the same range as the surgical techniques and can prevent the need for surgical staging procedures in up to 70% of cases [1,16,21]. The main aim of this study was to assess the utility of EUS-FNA for the diagnosis of enlarged MLNs and para-esophageal masses in the routine activity of a high-volume oncological center. The secondary aim was to assess the accuracy of this procedure according to the mass position. Then, we were to divide the patients based on the final diagnosis and to analyze the accuracy of EUS-FNA for each subgroup.

## 2. Materials and Methods

### 2.1. Study Design

We retrospectively reviewed the diagnostic performance of EUS-FNA in a cohort of patients who were referred to our oncological center between January 2019 and May 2020. The data analysis was performed with SPSS software (Version 23.0IBM SPSS Statistics, IBM Corporation, Chicago, IL, USA), the median and interquartile ranges were calculated for numerical variables, and the frequencies and percentages were reported for categorical factors. The sensitivity, specificity and accuracy were calculated according to positive predictive value (PPV) and negative predictive value (NVP).

### 2.2. Pre-Workup Evaluation

We evaluated all consecutive patients with enlarged or PET-positive MLNs (including patients with suspected lung cancer, suspected extra-thoracic neoplasia or lymphoma or other pathologies) and patients with para-mediastinal masses. All patients with suspicious mediastinal malignancies were discussed with in our thoracic endoscopy unit composed of a thoracic surgeon, thoracic endoscopist, dedicated radiologists and pathologists. We investigated N2 stations and N1 stations, and we sampled all sonically evident MLNs. We used EUS-FNA in the case of enlarged MLNs in stations 4L, 5, 7, 8, 9 and 3P. In the other MLN stations, paratracheal stations (2,4) and hilum stations (10,11), EBUS-TBNA is indicated.

All the patients eligible for sampling with EUS-FNA had enlarged MLNs or para-mediastinal masses evaluated based on recent (within 2–3 months) neck, thorax and upper abdomen CT scans and/or FDG PET.

According to the oncological lung guidelines, potentially pathological (defined abnormal) MLNs are represented by those, observed in a CT scan, with short axis diameters over 1 cm and/or positive fluorodeoxyglucose FDG-PET activity with a standard uptake value of 2 or greater. The prior diagnostic evaluation included conventional work-up with medical history, physical examinations, laboratory tests and bronchoscopy.

### 2.3. Endoscopic Ultrasound Technique

EUS-FNA was performed in order to obtain a histologically proven diagnosis of cancer and to acquire an accurate staging of the disease by confirming the involved lymph nodes.

Endoscopic ultrasound was performed with a dedicated linear echo-endoscope (Olympus GF-UCT 140) that was connected to the ultrasound unit (ALOKA Alpha 10). The procedures were performed under conscious sedation with intravenous infusion of midazolam (2–5 mg ev) and meperidine (10–30 mg ev) immediately before the procedures with accurate monitoring of patient parameters. Local oropharynx anesthesia was obtained by using 1% lidocaine spray and gel.

The echo-endoscope was initially introduced up to the level of the coeliac axis and was gradually withdrawn upwards for a detailed mediastinal imaging. A systematic examination of every mediastinal lymph node station was achieved. We examined by EUS the lower mediastinal nodes (stations 8 and 9), nodes of the aorto-pulmonary window (station 5), paratracheal nodes (stations 2L, 2R, 4L and 4R), retrotracheal nodes (station 3P) and subcarinal nodes (station 7).

When a primary lung tumor was accessible, an EUS-FNA on the lung mass was performed directly. Systematic measurements of all nodes were taken.

**Figure 1:**
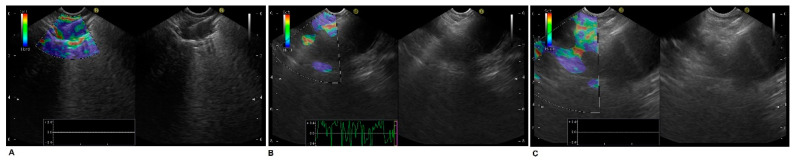
EUS-FNA lung cancer staging procedure with elastography: (**A**) Right lower lobe NSCLC. (**B**) Station 7. (**C**) Station 5.

EUS-FNA was performed by well-trained operators in an outpatient setting with an observation time after the procedures, enduring approximately 6 h. Pulse and color Doppler ultrasonography imaging were used to avoid vascular structures during the insertion of the needle. We routinely used a 22-gauge needle (Cook EchoTip^®^, Cook Medical, IN, USA). Sometimes, we utilized a 19-gauge needle or a 22-gauge procore needle (Cook EchoTip^®^, Cook Medical, IN, USA). The aspiration technique was used in all cases. After introducing the needle into the lesion, the stylet was gently removed from the needle, and a syringe was attached, allowing negative pressure to aspirate cells; the needle was moved within the target lesion back and forth about fifteen to twenty times. We did not use the slow-pull technique because the diagnostic yield of this technique is still under debate. Figure 1 reports a paraoesophageal right lung tumor EUS-FNA with mediastinal LN staging.

### 2.4. Cytology and Histology Preparation Method

In absence of an on-site cytologist, three to six needle passes were performed for each lesion to increase diagnostic yield. We would like to emphasize that the first evaluation of the tissue sampling adequacy was evidenced by the presence of a long strain of tissue (>1 cm) in the test tube, without the presence of a large amount of blood. The samples were collected in ThinPrep for cytology and were placed into formalin for histological examination. For the ThinPrep method, the samples were directly instilled into a single vial containing a liquid-based fixation medium for ThinPrep processing. The ThinPrep smear was stained with Papanicolaou stain using a routine laboratory protocol. One cytopathologist who was experienced in assessing smear slides and ThinPrep slides classified the findings.

## 3. Results

A total of 208 EUS-FNA were performed in 197 consecutive patients with a median age of 65 years (range 24–87 years) from January 2019 to May 2020. The general characteristics of the patients are reported in Table 1.

**Figure 2:**
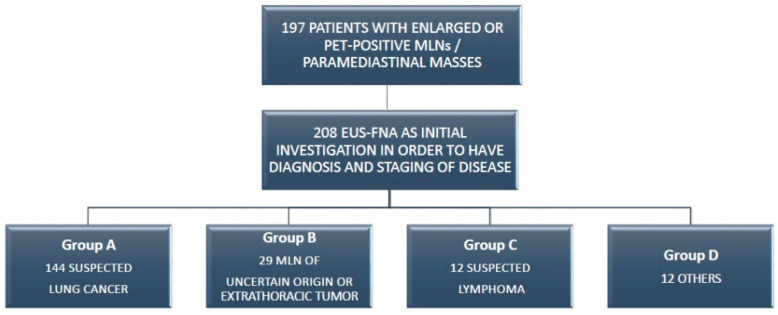
Distribution of patients in which EUS-FNA was performed according to suspected or previous pathology.

We considered four groups of patients according to different diseases (Figure 2).

Group A patients consisted of 147 patients with suspected lung cancer. EUS-FNA was directly performed on the pulmonary mass in 29 patients, on abnormal MLNs in 106 patients and on both sites in 9 patients. Group B consisted of 29 patients with an active or previous diagnosis of an extrathoracic malignancy and an abnormal MLN without evidence of a primary lung tumor. EUS-FNA was carried out on the MLNs. Group C consisted of 12 patients with suspected lymphoma. In the last 12 patients (group D), EUS-FNA was performed in order to confirm the diagnosis of other various diseases. EUS-FNA was performed at the following mediastinal sites according to the regional lymph node map definitions: at station 7 in 114 cases; at stations 8 and 9 in 4 cases; at station 3P and 4L in 1 and 13 cases, respectively; and at station 5 in 14 cases. Some patients had EUS-FNA performed in more than one lymph node station. In 2 cases, we sampled station 4L and 5 in the same procedure; stations 7 and 8/9 in 5 cases; and stations 4L and 7 in 1 case. The results according to the nodal station involvement are shown in Table 2.

In 34 cases, FNA was directly performed on the masses (32 para-mediastinal masses). In 9 cases, we sampled both para-mediastinal masses and station 7 lymph nodes.

### Diagnostic Features

**Figure 3:**
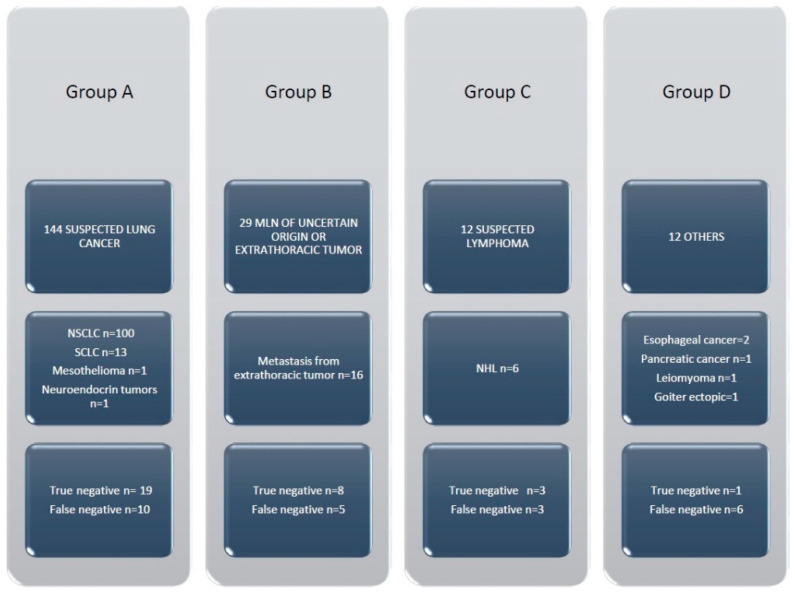
Final diagnoses in the four groups of patients.

The final diagnosis of the Group A patients (n = 144) included: NSCLC (n = 100, 69.4%), small cell lung cancer (SCLC) (n = 13, 9.0%), a neuroendocrine tumor (n = 1, 0.7%) and mesothelioma (n = 1, 0.7%). In 29 cases, we had negative diagnoses (no tumor cells), and in 19 cases, the specimens were adequate (negative diagnosis was confirmed by surgery). In 10 cases, the specimens were considered not adequate (amorphous material and fibrosis), and surgery was necessary to obtain the final diagnosis of NSCLC in 9 cases and mesothelioma in 2 cases. The final diagnoses of the group B patients (n = 29) were metastases from breast cancer (n= 2), colon cancer (n= 2), renal cancer (n = 3), prostate carcinoma (n = 1), liver cancer (n = 1), gastric cancer (n = 1), melanoma (n = 2), oropharyngeal squamous carcinoma (n = 1) and bladder cancer (n = 3). There were negative diagnoses in 13 cases (no tumor cells). Adequate specimens and negative diagnoses were confirmed by surgery in 8 cases. The specimens were considered not adequate for diagnosis, and surgery was required to obtain a malignant diagnosis in 5 patients. The final diagnoses of the group C patients (n = 12) with suspicion of lymphoma were non-Hodgkin lymphoma (NHL) in 6 cases (50%). In 3 patients, negative diagnoses were confirmed by surgery. In 3 patients, samples were false negative, and surgery was necessary to obtain the final diagnosis of NHL. In the last 12 patients (group D), other diseases were diagnosed in 5 cases. In 6 cases, surgery was performed to obtain a diagnosis of sarcoidosis (n = 5) and thymic carcinoma (n = 1), and 1 case of true negative was confirmed (Figure 3).

The diagnostic yield according to the different sites of the FNA is reported in Table 2.

The more frequent sites of biopsy were stations 7–8, with an accuracy of 84.4%. The overall sensitivity of EUS-FNA was 85%, with a corresponding negative predictive value (NPV) of 56% and an accuracy of 87.5%. In patients with abnormal MLNs, the sensitivity and accuracy of EUS-FNA was 81.1% and 84.4%, respectively, with NPV of 52.1%.

The sensitivity, accuracy and NPV of EUS-FNA for the detection of malignancies according to pathology groups is reported in Table 3. In group A, a higher value of sensitivity was recorded in the subcarinal–paraoesophageal stations, and in station 5, the sensitivity was 87.5% with an accuracy of 87.5%. In group B, the sensitivity of EUS-FNA at stations 7-8-9 was 80% with an NPV of 62.5%. The sensitivity of station 4L was 50% with an NPV of 66% and an accuracy of 75%. Finally, station 5’s sensitivity was 75% with an NPV of 50% and an accuracy of 80%.

Complications occurred in one patient, who was referred to our hospital for running a fever two days after the procedure. The patient underwent chest CT scans that showed a lymph node abscess. Then, a wide spectrum of antibiotics therapy was set, and the patient was discharged after seven days without signs of inflammation.

**Figure 4:**
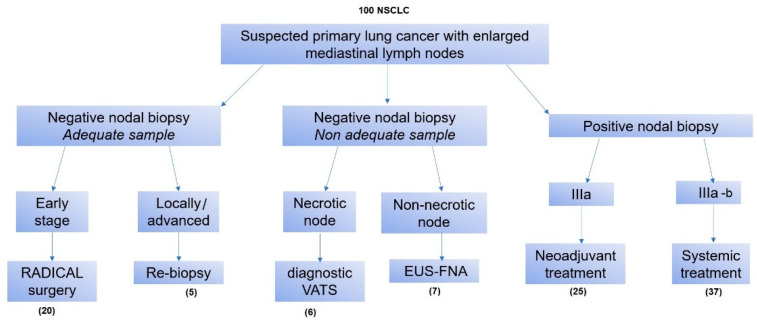
Flow chart of the management of suspected lung cancer with enlarged mediastinal lymph nodes.

The management of the patients with suspected lung cancer with enlarged mediastinal lymph nodes was resumed, as shown in Figure 4.

In 20 patients of group A with suspected locally advanced lung cancer (clinical stage IIIa) and with EUS-FNA on MLNs that resulted negative with adequate specimens, the patients were down-staged, and we confirmed the diagnosis after radical surgery. Indeed, patients were admitted in our thoracic surgery department and were scheduled for minimally invasive radical surgery. After an intraoperative frozen section on the primary tumor, in the case of the presence of NSCLC, we performed anatomical major lung resection and radical lymphadenectomy. Histological examinations after surgery confirmed the NSCLC on lung lesions and the absence of tumor cells on mediastinal lymph nodes in all patients. For 25 patients of the other groups (B, C, D) with no evidence of tumor cells in EUS-FNA specimens, minimally invasive surgery was needed. The patients underwent VATS in order to perform mediastinal lymphadenectomy and to obtain adequate specimens for histological examination.

## 4. Discussion

Before the advent of EBUS-TBNA and EUS-FNA, mediastinoscopy had been the mainstay for investigating abnormal MLNs [22]. This procedure requires general anesthesia and hospitalization and even though mortality in experienced centers is negligible, and morbidity rates may exceed 5% [23,24].

EUS-FNA provides a minimally invasive alternative for surgical staging, as it can prevent about 70% of scheduled mediastinoscopies [25,26]. As a result of its complementary diagnostic reach, the addition of EUS to mediastinoscopy improves staging and therefore reduces the number of futile thoracotomies [27]. EUS-FNA can also be used for mediastinal staging for extrathoracic tumors [28].

Lymph node metastases have an impact on prognosis and choice of therapy [29,30,31]. A correct mediastinal lymph node staging refers not only to making a difference between N0, N1, N2 or N3 diseases but also to making a careful assessment of the extent of lymph node metastasis (single station versus multiple stations) [32,33].

In a prospective study of 60 patients, the sensitivity of EUS-FNA in the right paratracheal lymph node station (4R) was 67% versus 33% of mediastinoscopy. At station 4L, EUS-FNA was also more sensitive than mediastinoscopy (80% vs. 33%). The sensitivity of EUS-FNA at station 7 was 100% versus only 7% for mediastinoscopy. The results of the yield for mediastinoscopy in this study are much lower than what has been reported in the past; therefore, EUS-FNAB seems to be the modality of choice [34].

Talebian and colleagues in 2009 reported an overall sensitivity, NPV and accuracy of EUS-FNA for N2/N3 disease to be 74%, 73% and 85% [35].

In 2013, Zielinsky reported primary staging in NSCLC patients with a sensitivity of EUS-FNA of 87.8% with an NPV of 82.5%, compared to a sensitivity of 64.3% in restaging the disease after neoadjuvant treatment. They concluded that EUS-FNA is the standard procedure for the primary staging of NSCLC [36].

Cerfolio et al. assessed the efficacy of different techniques of lymph node biopsies in patients with suspected metastatic NSCLC in station 5 and station 6 lymph nodes. They evaluated 112 patients clinically staged with N2 disease at station 5 or 6, and all were pathologically staged by mediastinoscopy, EUS FNAB, left uniportal VATS or anterior mediastinotomy. EUS-FNAB was used to biopsy suspicious aortopulmonary window (#5), subcarinal (#7), periesophageal (#8) and inferior pulmonary ligament (#9) lymph nodes. VATS was used in this study to sample the 5, 6, 7, 8 and 9 lymph nodes on the left, and the Chamberlain procedure was used to sample stations 5 and 6. Their conclusion was that, in N2 disease with the involvement of 5 and/or 6 suspicious lymph nodes, the left VATS staging procedure was preferred to prove or disprove cancer because it was easier and less invasive to obtain these nodes compared with the Chamberlain procedure. They considered EUS-FNA an unreliable method to assess nodes 5 and 6, but they were accurate when used to assess the 4L lymph node station [37].

Cerfolio et al. described an overall sensitivity of EUS-FNA for the detection of N2 disease of 63%, an NPV of 80% and an accuracy of 85%. At lymph node stations 4R and 4L, they reported a sensitivity of 40%, an NPV of 91% and an accuracy of 92%. At lymph node stations 7, 8 and 9, sensitivity was 81%, NPV was 91% and accuracy was 93%. In the aortopulmonary window, station 5’s sensitivity was 50%, with an NPV of 83% and an accuracy of 88% [38].

In our report, we describe and discuss the reach and limits of endoscopic ultrasound in the approach of thoracic masses and lymph nodes according to the new node site definition of the IASLC thoracic lymph node map [39].

We focused our attention on the accuracy of EUS-FNA for mediastinal lymph node stations 4L and 5. Endosonographers, surgeons and radiologists often may call a specific lymph node 4L or 5 and assign it a different number. This represents a major problem in any study discussing this issue, as the same vocabulary must be used.

Station 4 lymph nodes are located paratracheally but are situated caudal to the transverse aortic arch plane. The sagittal plane on the left side of the trachea is the margin between the left and right. Station 5 lymph nodes are situated laterally to station 4L nodes, with the ligamentum arteriosum as an anatomic border. Station 4L nodes are situated caudal to the superior border of the aortic arch, and station 5 nodes are located caudal to the inferior border of the aortic arch [40,41,42].

Tournoy et al. stated that ligamentum arteriosum cannot be discerned by means of ultrasound, and the differentiation between 4L and 5 can be difficult to discern, especially when both stations contain suspect lymph nodes [43].

We think that, in some cases, the concomitant presence of 4L and 5 lymphadenopathies can visualize a thin hyperechoic band between the aortic arch and left pulmonary artery by EUS that is suggestive to be the ligamentum arteriosum (this is confirmed by the tent effect during the needle puncture).

Our results show that the overall diagnostic accuracy of EUS-FNA in this series is 87% with a sensitivity of 84.5%, comparable with those presented in other publications.

In stations 7, 8 and 9, sensitivity was 82.1%, and accuracy was 87.5%. In station 4L and station 5, sensitivity was 70% and 80%, respectively, with accuracies of 78.5% and 81.2% (Table 1).

Our results show a good level of accuracy and sensitivity of EUS-FNA both in subcarinal stations (n°7, 8 and 9) and at the 4L-5 lymph nodes stations. The low size of the 4L-5 groups (30 patients) limits these results and makes these two station groups not statistically comparable. However, the accuracy rates of the 4L-5 groups are higher than what is reported in literature, despite a relatively large number of station 5 lymph nodes (16/30; 52%). We report a higher accuracy and sensitivity of EUS-FNA at station 5 lymph nodes than other studies. We believe that endosonographs can visualize and biopsy station 5 lymph nodes through EUS with accuracy when the size and the location of the lymphadenopathy in the aorto-pulmonary station permits to open the window towards the oesophagus as well. This can occur with or without concomitant 4L lymphoadenopathy.

One limitation to our results is the high false negative rate with a corresponding low NPV. This value improved once we analyzed the specific pathology groups (groups A and B). The higher false negative rates of groups C and D depend on specific diseases, including lymphoma and sarcoidosis. The diagnosis of lymphoma with a non-invasive technique has a significant number of false negative findings in the cytology. Tests for the specific differential diagnosis of lymphoma include immunohistopathological examinations, so histological sampling is the preferred choice. In our routine practice, there was not the presence of an on-site cytologist, and the rapid onset evaluation (ROSE) was not usually performed. Despite the ROSE technique being considered one of the best methods to increase the accuracy of FNA, we reached high-quality results in terms of sensitivity and accuracy without ROSE. Indeed, we usually considered a sample adequate when there was a long strain of tissue (>1 cm) in the test tube, without the presence of a large amount of blood. Then, in case of suspected lymphoma [44,45,46].

The diagnosis of sarcoidosis requires specific testing for evidence of the presence of granulomas in the sample; therefore, specific technical expertise for the diagnosis of sarcoidosis is necessary [47,48,49].

Our study shows that, in patients with suspected lung cancer in whom mediastinal lymph node exploration is required, EUS-FNA reduces the need of surgical staging procedures. As shown in our results, EUS-FNA allows for the complete staging of the mediastinum, including the sampling of station 5. Our dedicated group for the diagnosis of thoracic diseases was composed of a radiologist, thoracic surgeon, thoracic endoscopist and pathologist, and it was crucial to perform a quick and accurate diagnosis and to allow us to give patients the most appropriate treatment in relation to their thoracic disease.

## Figures and Tables

**Table 1:** Patients and procedure characteristics.

Group	Group A	Group B	Group C	Group D
**Age**	65 (37–82)	67 (29–80)	49 (24–65)	54 (27–79)
**Gender (M/F)**	96/48	17/12	8/4	7/5
**ECOG score**	3 (2–4)	3 (2–4)	2 (1–4)	3 (2–5)
**Median diameter of puncture target**	23 (12–45)	19 (13–40)	25 (17–55)	22 (11–39)
**Needle size (22/19)**	112/35	20/9	4/8	8/4
**Number of punctures**	4 (3–6)	3 (2–6)	4 (3–6)	4 (3–6)
**Perioperative complications**	1	0	0	0

**Table 2:** Diagnostic yield according to the site of EUS-FNA biopsy.

Site of the Biopsy	Number of Patients	Sensitivity	Negative Predictive Value	Diagnostic Accuracy
**Overall MLNs**	154	81.1%	52.1%	84.4%
**Stations 7 + 8 + 9**	123	82.1%	55%	87.5%
**Station 4L**	14	70%	57.1%	78.5%
**Station 5**	16	80%	25%	81.2%
**Station 3P**	1	100%	0	81.2%
**Lung Mass**	32	96.4%	80%	96.8%
**Lung Mass and MLN**	9	100%	NA	100%
**Others**	2	100%	NA	100%

**Table 3:** Sensitivity, NPV and diagnostic accuracy of lymph node stations by pathology group.

Pathology Group	Stations 7, 8 and 9 (Sensitivity, NPV, Accuracy)	Station 4L (Sensitivity, NPV, Accuracy)	Station 5 (Sensitivity, NPV, Accuracy)
Group A	90.5%; 66%; 92%	75%; 33%; 77%	87.5%; NC; 87.5%
Group B	80%; 62.5%; 83.3%	50%; 66%; 75%	75%; 50%; 80%
Group C	57.1%; 40%; 66%	NA; 100%; 100%	100%; NA; 100%
Group D	0%; 16.6%; 16.6%	NA; NA; NA	0%; 0%; 0%

## Data Availability

The data are available on request.

## References

[1] Dhooria S., Mehta R.M., Madan K. (2019). A Multicenter Study on the Utility of EBUS-TBNA and EUS-B-FNA in the Diagnosis of Mediastinal Lymphoma. J. Bronchol. Interv. Pulmonol..

[2] Colella S., Scarlata S., Bonifazi M., Ravaglia C., Naur T.M.H., Pela R., Clementsen P.F., Gasparini S., Poletti V. (2018). Biopsy needles for mediastinal lymph node sampling by endosonography: Current knowledge and future perspectives. J. Thorac. Dis..

[3] Sakairi Y., Nakajima T., Yoshino I. (2019). Role of endobronchial ultrasound-guided transbronchial needle aspiration in lung cancer management. Expert Rev. Respir. Med..

[4] Youlden D.R., Cramb S.M., Baade P.D. (2008). The International Epidemiology of Lung Cancer Geographical Distribution and Secular Trends. J. Thorac. Oncol..

[5] Nanavaty P., Alvarez M.S., Alberts W.M. (2014). Lung cancer screening: Advantages, controversies, and applications. Cancer Control.

[6] Gridelli C., Rossi A., Carbone D.P., Guarize J., Karachaliou N., Mok T., Petrella F., Spaggiari L., Rosell R. (2015). Non-small-cell lung cancer. Nat. Rev. Dis. Primers.

[7] Jacobsen M.M., Silverstein S.C., Quinn M., Waterston L.B., Thomas C.A., Benneyan J.C., Han P.K.J. (2017). Timeliness of access to lung cancer diagnosis and treatment: A scoping literature review. Lung Cancer.

[8] Lee J.W., Kim E.Y., Kim D.J., Lee J.H., Kang W.J., Lee J.D., Yun M. (2016). The diagnostic ability of 18F-FDG PET/CT for mediastinal lymph node staging using 18F-FDG uptake and volumetric CT histogram analysis in non-small cell lung cancer. Eur. Radiol..

[9] Schmidt-Hansen M., Baldwin D.R., Hasler E., Zamora J., Abraira V., Roqué I Figuls M. (2014). PET-CT for assessing mediastinal lymph node involvement in patients with suspected resectable non-small cell lung cancer. Cochrane Database Syst. Rev..

[10] Yin Z., Liang Z., Li P., Wang Q. (2017). CT-guided core needle biopsy of mediastinal nodes through a transpulmonary approach: Retrospective analysis of the procedures conducted over six years. Eur. Radiol..

[11] Groheux D., Quere G., Blanc E., Lemarignier C., Vercellino L., de Margerie-Mellon C., Merlet P., Querellou S. (2016). FDG PET-CT for solitary pulmonary nodule and lung cancer: Literature review. Diagn Interv. Imaging.

[12] Vegar Zubović S., Kristić S., Hadžihasanović B. (2017). Positron emission tomography/computed tomography (PET/CT) and CT for N staging of non-small cell lung cancer. Med Glas.

[13] Gao S.J., Kim A.W., Puchalski J.T., Bramley K., Detterbeck F.C., Boffa D.J., Decker R.H. (2017). Indications for invasive mediastinal staging in patients with early non-small cell lung cancer staged with PET-CT. Lung Cancer.

[14] De Leyn P., Dooms C., Kuzdzal J., Lardinois D., Passlick B., Rami-Porta R., Turna A., Van Schil P., Venuta F., Waller D. (2014). Preoperative mediastinal lymph node staging for non-small cell lung cancer:2014 update of the 2007 ESTS guidelines. Transl. Lung Cancer Res..

[15] Rendina E.A., Venuta F., De Giacomo T., Ciccone A.M., Moretti M.S., Ibrahim M., Coloni G.F. (2002). Biopsy of anterior mediastinal masses under local anesthesia. Ann. Thorac. Surg..

[16] Witte B., Neumeister W., Huertgen M. (2008). Does endoesophageal ultrasound-guided fine-needle aspiration replace mediastinoscopy in mediastinal staging of thoracic malignancies?. Eur. J. Cardio-Thorac. Surg..

[17] Trosini-Désert V., Jeny F., Maksud P., Giron A., Degos V., Similowski T. (2019). Contribution of endobronchial ultrasound elastography to the characterization of mediastinal lymphadenopathy: A single-center, prospective, observational study. Respir. Med. Res..

[18] Tournoy K.G., Praet M.M., Van Maele G., Van Meerbeeck J.P. (2005). Esophageal endoscopic ultrasound with fine-needle aspiration with on-site pathologist:high accuracy for the diagnosis of mediastinal lymphadenopathy. Chest.

[19] Sanchez-Lorente D., Guzman R., Boada M., Guirao A., Carriel N., Molins L. (2018). N2 disease in non-small-cell lung cancer: Straight to surgery?. Future Oncol..

[20] Boffa D., Fernandez F.G., Kim S., Kosinski A., Onaitis M.W., Cowper P., Jacobs J.P., Wright C.D., Putnam J.B., Furnary A.P. (2017). Surgically Managed Clinical Stage IIIA-Clinical N2 Lung Cancer in The Society of Thoracic Surgeons Database. Ann. Thorac. Surg..

[21] Annema J.T., Versteegh M.I., Veseliç M., Voigt P., Rabe K.F. (2005). Endoscopic ultrasound-guided fine-needle aspiration in the diagnosis and staging of lung cancer and its impact on surgical staging. J. Clin. Oncol..

[22] Toloza E.M., Harpole L., Detterbeck F., McCrory D.C. (2003). Invasive staging of non-small cell lung cancer: A review of the current evidence. Chest.

[23] Hujala K.T., Sipila J.L., Grenman R. (2001). Mediastinoscopy: Its role and value today in the differential mediastinal pathology. Acta Oncol..

[24] Sehgal I.S., Dhooria S., Aggarwal A.N., Behera D., Agarwal R. (2016). Endosonography Versus Mediastinoscopy in Mediastinal Staging of Lung Cancer: Systematic Review and Meta-Analysis. Ann. Thorac. Surg..

[25] Sharples L.D., Jackson C., Wheaton E., Griffith G., Annema J.T., Dooms C., Tournoy K.G., Deschepper E., Hughes V., Magee L. (2012). Clinical effectiveness and cost-effectiveness of endobronchial and endoscopic ultrasound relative to surgical staging in potentially resectable lung cancer: Results from the ASTER randomized controlled trial. Health technol Assess. Health Technol. Assess..

[26] Gallina F.T., Assisi D., Forcella D., Pierconti F., Visca P., Melis E., Facciolo F. (2021). Five years of thoracic endoscopy unit activity on lung cancer staging: How teamwork can improve the outcomes. Mediastinum.

[27] Larsen S.S., Vilmann P., Krasnik M., Dirksen A., Clementsen P., Maltbaek N., Lassen U., Skov B.G., Jacobsen G.K. (2005). Endoscopic ultrasound guided biopsy performed routinely in lung cancer staging spares futile thoracotomies: Preliminary results from a randomized clinical trial. Lung Cancer.

[28] Kramer H., Koëter G.H., Sleijfer D.T., van Putten J.W., Groen H.J. (2004). Endoscopic ultrasound-guided fine-needle aspiration in patients with mediastinal abnormalities and previous extrathoracic malignancy. Eur. J. Cancer.

[29] Woodard G.A., Jones K.D., Jablons D.M. (2016). Lung Cancer Staging and Prognosis. Cancer Treat Res..

[30] Gamliel Z. (2016). Mediastinal Staging in Non-Small Cell Lung Cancer. Surg. Oncol. Clin. N. Am..

[31] Abdel-Rahman O. (2018). Validation of the AJCC 8th lung cancer staging system among patients with small cell lung cancer. Clin. Transl. Oncol..

[32] Keller S.M., Vangel M.G., Wagner H., Schiller J.H., Herskovic A., Komaki R., Marks R.S., Perry M.C., Livingston R.B., Johnson D.H. (2004). Prolonged survival in patients with resected non-small cell lung cancer and single-level N2 disease. J. Thorac. Cardiovasc.Surg..

[33] Okada M., Tsubota N., Yoshimura M., Miyamoto Y., Matsuoka H. (1999). Prognosis of completely resected pN2 non-small cell lung carcinomas: What is the significant node that affecys survival?. J. Thorac. Cardiovasc. Surg..

[34] Larsen S.S., Vilmann P., Krasnik M., Dirksen A., Clementsen P., Skov B.G., Jacobsen G.K. (2005). Endoscopic ultrasound guided biopsy versus mediastinoscopy for analysis of paratracheal and subcarinal lymph nodes in lung cancer staging. Lung Cancer.

[35] Talebian M., von Bartheld M.B., Braun J., Versteegh M.I., Dekkers O.M., Rabe K.F., Annema J.T. (2010). EUS –FNA in the preoperative staging of non-small cell lung cancer. Lung Cancer.

[36] Zielinski M., Szlubowski A., Kołodziej M., Orzechowski S., Laczynska E., Pankowski J., Jakubiak M., Obrochta A. (2013). Comparison of endobronchial ultrasound and/or endoesophageal ultrasound with transcervical extended mediastinal lymphadenectomy for staging and restaging of Non-Small Cell Lung Cancer. J. Thorac. Oncol..

[37] Cerfolio R.J., Bryant A.S., Eloubeidi M.A. (2007). Accessing the aortopulmonary window (#5) and the paraaortic (#6) lymph nodes in patients with Non-Small Cell Lung Cancer. Ann. Thorac. Surg..

[38] Cerfolio R.J., Bryant A.S., Eloubeidi M.A., Frederick P.A., Minnich D.J., Harbour K.C., Dransfield M.T. (2010). The true false negative rates of esophageal and endobronchial ultrasound in the staging of mediastinal lymph nodes in patients with Non-Small Cell Lung Cancer. Ann. Thorac. Surg..

[39] Rusch V.W., Asamura H., Watanabe H., Giroux D.J., Rami-Porta R., Goldstraw P. (2009). The IASLC Lung Cancer Staging Project: A proposal for a new international lymph node map in the forthcoming seventh edition of the TNM classification for lung cancer. J. Thorac. Oncol..

[40] Roth K., Eagan T., Hardie J. (2016). Expert opinion of mediastinal lymph node positions from an intrabronchial view. BMC Pulm. Med..

[41] Oliveira R.L., Liberman M. (2017). Endosonographic Mediastinal Lymph Node Staging in Non-Small Cell Lung Cancer: How I Teach It. Ann. Thorac. Surg..

[42] Carter B.W., Marom E.M., Detterbeck F.C. (2014). Approaching the patient with an anterior mediastinal mass: A guide for clinicians. J. Thorac. Oncol..

[43] Tournoy K.G., Annema J.T., Krasnik M., Herth F.J., van Meerbeeck J.P. (2009). Endoscopic and Endobronchial Ultrasonography According to the proposed lymph node map definition in the seventh edition of the tumor, node, metastasis classification for lung cancer. J. Thorac. Oncol..

[44] Creemers K., van der Heiden O., Los J., van Esser J., Newhall D., Djamin R.S., Aerts J.G. (2011). Endoscopic Ultrasound Fine Needle Aspiration in the Diagnosis of Lymphoma. J. Oncol..

[45] Yim A.P. (1995). Video-assisted thoracoscopic management of anterior mediastinal masses. Preliminary experience and results. Surg. Endosc..

[46] Agid R., Sklair-Levy M., Bloom A.I., Lieberman S., Polliack A., Ben-Yehuda D., Sherman Y., Libson E. (2003). CT-guided biopsy with cutting-edge needle for the diagnosis of malignant lymphoma: Experience of 267 biopsies. Clin. Radiol..

[47] Gupta D., Dadhwal D.S., Agarwal R., Gupta N., Bal A., Aggarwal A.N. (2014). Endobronchial Ultrasound.Guided Transbronchia Needle Aspiration vs Conventional Transbronchial Needle Aspiration in The Diagnosis of Sarcoidosis. Chest.

[48] Prasse A. (2016). The Diagnosis, Differential Diagnosis, and Treatment of Sarcoidosis. Dtsch. Arztebl. Int..

[49] Soto-Gomez N., Peters J.I., Nambiar A.M. (2016). Diagnosis and Management of Sarcoidosis. Am. Fam. Physician.

